# Recent Advances in Proximity Labeling-Based Subcellular Proteomic Mapping

**DOI:** 10.1016/j.mcpro.2026.101520

**Published:** 2026-01-29

**Authors:** Gang Wang, Jiapeng Liu, Xuege Sun, Wei Qin, Shuo Han, Peng Zou

**Affiliations:** 1 College of Chemistry and Molecular Engineering, Synthetic and Functional Biomolecules Center, Beijing National Laboratory for Molecular Sciences, Key Laboratory of Bioorganic Chemistry and Molecular Engineering of Ministry of Education, PKU-IDG/McGovern Institute for Brain Research, Beijing Advanced Center of RNA Biology (BEACON), Peking University, Beijing, China; 2 Key Laboratory of RNA Innovation, Science and Engineering, Shanghai Institute of Biochemistry and Cell Biology, Center for Excellence in Molecular Cell Science, Chinese Academy of Sciences, University of Chinese Academy of Sciences, Shanghai, China; 3 School of Pharmaceutical Sciences, Tsinghua-Peking Center for Life Sciences, MOE Key Laboratory of Bioorganic Phosphorus Chemistry & Chemical Biology, The State Key Laboratory of Membrane Biology, Beijing Frontier Research Center for Biological Structure, Tsinghua University, Beijing, China; 4 Academy for Advanced Interdisciplinary Studies, Peking-Tsinghua Center for Life Sciences, Peking University, Beijing, China; 5 Chinese Institute for Brain Research (CIBR), Beijing, China

**Keywords:** proximity labeling, functional PL, protein interactome, photocatalytic PL, antibody-targeted PL

## Abstract

The spatial organization of the cellular proteome is vital for cellular physiology, as protein localization is closely linked to post-translational modifications, subcellular trafficking, and protein-protein interactions. Systematic profiling of these spatial features can greatly enhance our understanding of protein functions. Recent advances in enzyme-mediated proximity labeling (PL) techniques, such as TurboID and APEX2, have improved our ability to map subcellular proteomes in living cells. This review discusses emerging trends in PL methods, which now offer subcellular precision with multi-dimensional protein features, including post-translational modifications, trafficking, turnover, and interaction with other biomolecules. Additionally, new techniques such as photoactivatable PL (optoPL) and antibody-targeted PL (immunoPL) provide enhanced spatiotemporal control and allow for detailed subcellular proteome mapping without genetic manipulation.

## Highlights

• Functional proximity labeling (PL) enables profiling protein features with subcellular precision.
• PL provides valuable insights into protein interactomes with diverse biomolecules.
• Photocatalytic PL allows enhanced spatiotemporal mapping of subcellular proteomes.
• Antibody-targeted PL facilitates subcellular proteome profiling without genetic manipulation.

## Graphical Abstract

**Figure:**
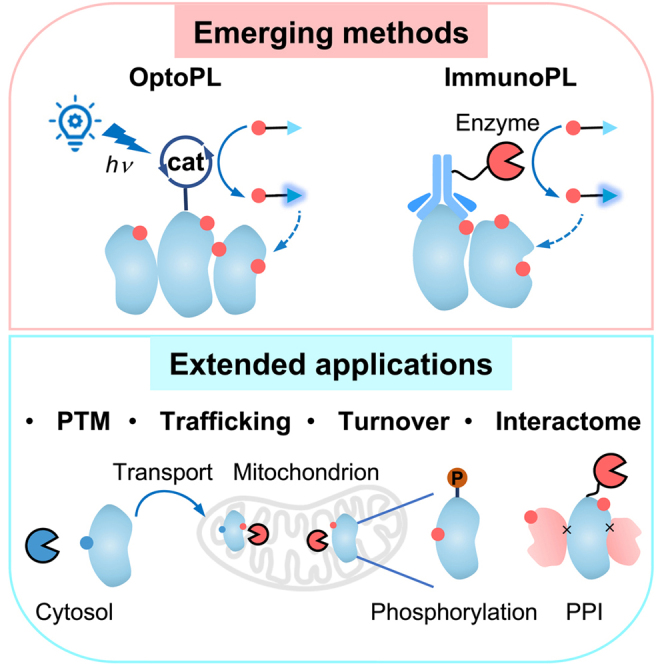

## In Brief

This review highlights recent advances in proximity labeling (PL) technologies, including their integration with complementary techniques to probe multidimensional protein features at subcellular resolution as well as the development of photoactivatable and antibody-targeted PL strategies.

Subcellular protein localization is crucial for its function, as it determines the specific tasks proteins carry out within distinct subcellular compartments. Protein localization is often tightly linked to protein features such as post-translational modifications (PTMs), trafficking, and protein-protein interactions, etc. For example, phosphorylation by protein tyrosine kinases often regulates the spatial distribution of protein substrates to initiate signaling pathways (1). The pattern of protein glycosylation is strongly correlated with protein compartmentalization in the secretory pathway and regulates protein folding during quality control (2, 3). Protein subcellular localization is regulated by trafficking, which allows for spatial and temporal control of cellular activities. Together, these mechanisms ensure the precise localization and activity of proteins, thereby maintaining cellular homeostasis and enabling diverse biological functions.

An array of techniques has been developed to map the landscape of subcellular proteome. Imaging techniques such as immunofluorescence microscopy and live-cell imaging enable the direct visualization of specific proteins within organelles and subcellular structures (4, 5). Despite their high spatial resolution, imaging techniques are often limited by their throughput. Recently invented multiplexed, high throughput imaging techniques, on the other hand, require sophisticated instrumentations that are beyond the reach of most labs (6, 7). Subcellular fractionation methods, including differential centrifugation and density gradient centrifugation, facilitate the isolation of organelles and subcellular fractions, allowing for mass spectrometry (MS)-based characterization of their content at the proteome level (8, 9). However, these fractionation-based methods often lack sufficient specificity. To address the above challenges, enzyme-mediated proximity labeling (PL) approaches, such as TurboID (10) and APEX2 (11, 12), have been developed to allow the identification of proteins proximal to a target of interest within living cells, providing insights into protein-protein interactions and subcellular localization *in situ*. These complementary methodologies collectively contribute to our understanding of subcellular protein dynamics and function, enabling the elucidation of complex cellular processes.

In this review, we highlight several emerging trends in PL technology development (Table 1). These innovative approaches offer the means to map protein features with subcellular precision, including PTMs, protein trafficking, turnover, protein-protein interactome, and nucleic acid-protein interactome. Additionally, we discuss recent techniques such as photoactivatable methods for enhanced spatiotemporal control of PL (optoPL) and antibody-targeted PL methods (immunoPL), which facilitate the mapping of the subcellular proteome without the need for genetic manipulation.

**Table 1:** Overview of PL methods

Methods	Description	Advantages	Limitations
Canonical PL			
Peroxidase-mediated PL (APEX, APEX2, HRP)	Peroxidase generates highly reactive free radicals triggered by H_2_O_2_ to label proximal biomolecules in living cells	High spatial and temporal resolution; versatility for protein, RNA, and DNA labeling	Limited *in vivo* application due to cytotoxicity of H_2_O_2_
Biotin ligase-mediated PL (BioID, BASU, TurboID)	Biotin ligase converts biotin to biotinyl-5′-AMP to react with lysine residue of nearby proteins	High spatial resolution; non-toxic for *in vivo* application	Presence of endogenous biotin compromises temporal resolution
Opto-PL			
PhotoTurbo	Photocleavable nitrobenzyl-caged catalytic lysine inhibits TurboID activity in the dark	Minimal background activity; enhanced temporal control compared to TurboID	Requirement of unnatural amino acid incorporation; phototoxicity of UV irradiation
LOV-Turbo	Photosensitive LOV domain distorts TurboID’s substrate-binding pocket in the dark	Minimal background activity; enhanced temporal control compared to TurboID; reversible compared to photoTurbo	Lower efficiency compared to TurboID; inactive in secretory pathway
MiniSOG-mediated PL (CAP-seq, RinID, PDPL, LITag)	Photosensitive protein miniSOG generates singlet oxygen upon blue light illumination to oxidize nearby biomolecules, which are captured with nucleophilic amine probes	High spatial and temporal resolution; versatility for protein, RNA, and DNA labeling	Cytotoxicity due to endogenous photosensitizers; limited tissue penetration of blue light
Synthetic organic chromophore-mediated PL (LUX-MS)	Photosensitive chromophores yield singlet oxygen or other active intermediates upon illumination to label proximal biomolecules	High singlet oxygen yield; convenient modification of chromophores	Requirement of additional strategies for subcellular targeting; non-specific absorption of dyes
Transition metal complex-mediated PL (μMap, μMap-Red)	Ir or Sn^IV^-chlorin complexes generate carbene or nitrene intermediates upon illumination to label proteins	Extremely high spatial resolution due to very short half-life of carbene	Requirement of additional strategies for subcellular targeting
CAT-Prox, CAT-S	Ir complex unmasks azidobenzyl-caged quinone methide (QM) for protein labeling	High biocompatibility with diverse live cell samples for mitochondrial proteome profiling	Long lifespan of QM intermediate; inherent mitochondrial accumulation of the Ir catalyst
Immuno-PL			
Antibody- or Protein A-guided PL (EMARS, BAR, AMAPEX)	Secondary antibody or Protein A are conjugated to PL enzymes for subcellular targeting in living cells and fixed samples	No requirement for genetic manipulation; versatility for diverse biological samples	Relying on antibody specificity; lack of cell specificity when applied to complex tissue samples
GFP nanobody-directed TurboID (BLITZ)	GFP nanobody (GBP) is conjugated to TurboID for targeting in GFP-expressing transgenic organisms	No requirement for creating transgenic models when mapping proteomes across various tissues *in vivo*	The binding of GBP-TurboID with GFP-tagged POI potentially perturbs the localization and function of the POI
Aptamer-conjugated peroxidase-mimicking DNAzyme	DNA aptamers targeting POI are coupled with hemin-binding DNAzyme for cell surface protein profiling	Convenient evolution and modification, and low production cost of the aptamer	Low labeling efficiency of DNAzyme; limited availability of specific aptamers

## Functional Proximity Labeling

The fusion of PL with functional protein enrichment strategies, termed "functional proximity labeling," represents a pivotal advancement in proteomics, offering a refined approach to investigate specific protein functional subclasses or proteoforms within subcellular compartments. This methodology allows for the targeted mapping of proteins with distinct functionalities while retaining the benefits of PL's unbiased protein discovery capabilities. By coupling PL with techniques such as affinity purification or activity-based labeling, specific protein subsets can be selectively captured and identified, providing comprehensive insights into protein localization, timing, and function. Functional PL thus represents a pivotal advancement in proteomic research, with implications for understanding cellular physiology, disease mechanisms, and the identification of potential therapeutic targets.

### Decoding Subcellular Post-Translational Modifications

PTMs, such as phosphorylation (13) and glycosylation (2), play a crucial role in finely tuning the function of the proteins they modify. Because regulatory enzymes governing PTMs exhibit distinct subcellular distributions, various types of PTMs display heterogeneous patterns across different cellular compartments (2, 14). Recent advancements in MS-based proteomics, coupled with targeted PTM enrichment strategies, have significantly advanced our understanding of PTM landscapes (15). Integrating PTM profiling with subcellular fractionation techniques enables the identification of PTM substrates within specific cellular locales (16, 17). For example, phosphorylated proteins within the mitochondria have been pinpointed by combining mitochondrial fractionation with phosphoprotein enrichment using immobilized metal affinity chromatography (IMAC) (17). However, biochemical fractionation techniques often face challenges in purifying subcellular regions, particularly non-membrane enclosed areas such as the nucleolus and stress granules, thereby hindering the analysis of functional proteoforms in these regions.

**Fig. 1:**
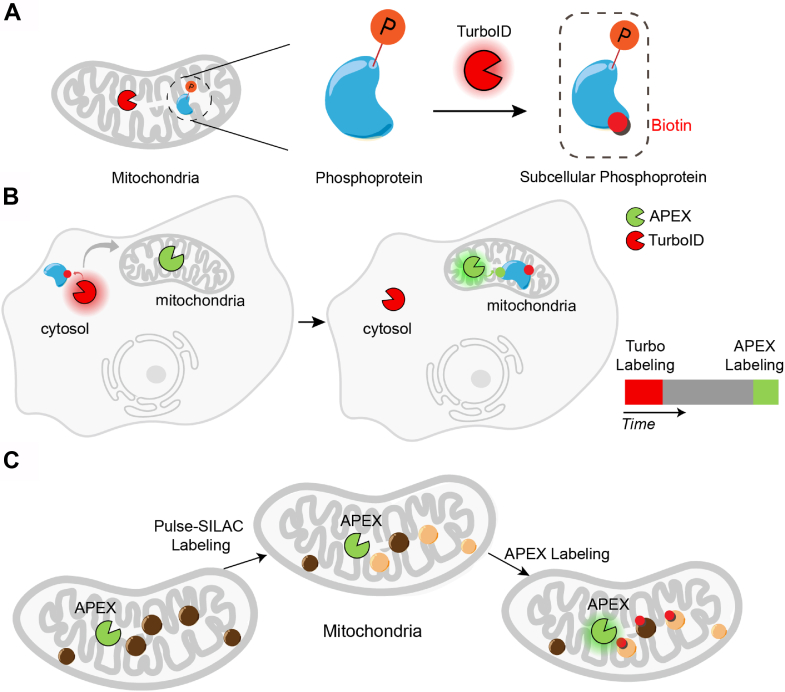
**Examples of functional PL**. *A*, the application of PL in mapping subcellular PTMs such as phosphorylation. *B*, dynamic mapping of protein trafficking using TransitID that multiplexes orthogonal PL enzymes. *C*, mapping subcellular protein turnover by prox-SILAC that integrates PL tools into the pulse-SILAC workflow.

Recent studies have capitalized on the spatial and temporal precision of PL alongside its compatibility with live-cell labeling to unravel PTMs at subcellular levels. Liu *et al.* combined TurboID with the phosphopeptide enrichment technique to achieve real-time monitoring of phosphorylation dynamics within the subcellular proteome of living cells and organisms (SubMAPP) (Fig. 1*A*) (18). SubMAPP unveiled new kinase substrates in the endoplasmic reticulum (ER) lumen and changes in protein phosphorylation during chemically induced ER stress. Similarly, a comparable strategy has been implemented by combining APEX labeling with the enrichment of phosphorylated or O-GlcNAcylated proteins (19). Moreover, beyond phosphorylation, TurboID has recently been fused with activity-based protein profiling (ABPP), particularly focusing on redox chemoproteomics, to monitor localized cysteine oxidation in live cells (20, 21). Intriguingly, the Weerapana laboratory demonstrated the activation of APEX by endogenously generated hydrogen peroxide, enabling biotinylation of proteins in close proximity to the source of reactive oxygen species (20).

Alternatively, PL has been harnessed to map PTM-dependent interacting proteins by introducing the PL enzyme or photocatalyst onto the PTM and labeling the surrounding proteins. The MacMillan lab pioneered the μMAP technology, utilizing an iridium-based photocatalyst to generate reactive carbene intermediates *via* Dexter energy transfer (22, 23). This approach was applied to introduce the iridium photocatalyst onto metabolically inserted azidosialic acid, facilitating the profiling of local microenvironments across the sialylated proteome (23). In a parallel endeavor, the Huang laboratory engineered an APEX2-fused protein with a glycan-binding domain, enabling targeting of specific glycan structures (24, 25, 26). This strategy led to the discovery of glycan-mediated glycoprotein interactors of galectin-1 and galectin-3 in living cells. Similarly, by fusing miniTurbo with the GlcNAc-binding lectin GafD, Liu *et al*. devised GlycoID to map O-GlcNAc-dependent interacting proteins in the nucleus and cytosol, respectively (27). Additionally, SUMO-ID was developed utilizing complementation-based proximity biotinylation with split-TurboID to uncover SUMO-dependent interactors of proteins of interest (28). These cutting-edge tools offer unprecedented insights into PTM-mediated functional protein hubs within living systems.

### Mapping Protein Trafficking

Protein localization is meticulously regulated and structured to uphold the division of biological processes (29, 30). The misplacement of proteins is closely linked to diseases, including cancer (31) and neurodegenerative disorders (32). Understanding protein trafficking is thus crucial for comprehending cell biology and developing therapeutic interventions for diseases. Protein trafficking encompasses intracellular protein transport within organelle (33, 34, 35), as well as extracellular transit between different organs (36). Various methodologies have been devised and implemented to tackle specific challenges in this field. Strategies such as fluorescent imaging (37) and affinity purification coupled with MS-based proteomics (38) have shed light on numerous critical factors influencing protein localization. However, none of these techniques can determine the specific organelle origin along the trafficking pathway to its destination in a systematic fashion.

A substantial number of proteins can be secreted into the extracellular space and achieve long-distance signal communications (36). Several studies have achieved *in vivo* protein trafficking by the integration of PL into the label-and-fractionation concept (39, 40, 41, 42, 43). These studies employed a similar methodology, wherein a selective biotin ligase was directed to the ER lumen of specific cell types *in vivo*. Subsequently, the process began with the labeling of secreted proteins using exogenous biotin. Once the proteins had completed their transit to the designated cellular destinations, where biotinylated proteins were captured *via* streptavidin enrichment. In addition, intracellular protein trafficking between organelles has been also mapped by PL in the source and subsequent recovery from the fractionated destination organelle.

Capturing the precise intracellular and intercellular trafficking of proteins presents significant challenges due to limitations in fractionation techniques, particularly with membraneless condensates and specific cell types like neurons and astrocytes. The barrier was shattered by the emergence of TransitID (44), an innovative solution by placing orthogonal PL enzymes on different organelles to obtain temporal and spatial information. In this TransitID workflow, TurboID is selectively tagged to the source compartment for its non-toxicity and activated *via* biotin exposure, followed by a customizable chase period to facilitate protein translocation to the target site. Subsequent APEX2 labeling at the destination, using an alkyne-phenol substrate, ensures proteins carry both TurboID and APEX2 labels, unequivocally originating from the source and completing translocation within the specified chase duration (Fig. 1*B*). TransitID offers exceptional adaptability, successfully mapping protein transport events between various organelles and cell types, such as stress granules and the nucleolus, as well as co-cultured cancer cells and macrophages, showcasing its extensive potential applications.

### Quantifying Protein Turnover Rates

For precise modulation of biological activity over time, proteins undergo continuous updates that are tightly regulated to ensure accurate renewal. Various strategies have made significant strides in measuring protein turnover dynamics at the whole-cell level. Typically, newly synthesized proteins are labeled using chemical tools such as non-canonical amino acids (45) or stable isotopes (46, 47). Dieterich *et al.* discovered hundreds of differentially regulated proteins by employing Bioorthogonal non-canonical amino acid tagging (BONCAT) at hippocampal synapses (48). Other studies have utilized stable isotope labels (pulse-SILAC) in cellular systems or even *in vivo*, elucidating the assembly kinetics of various biomolecular complexes (49, 50, 51, 52, 53, 54). However, these approaches are restricted to the whole-cell proteome level and lack spatial information within cells. To address this limitation, Buchwalter *et al.* and Bogenhagen *et al.* combined pulse-SILAC labeling with organelle purification workflows to measure protein turnover at subcellular resolution (55, 56, 57), yet still grapple with the challenges of error-prone and limited organelle purification methods.

To tackle these challenges, the Zou lab endeavored to integrate PL tools into the pulse-SILAC workflow, resulting in the development of prox-SILAC (58). In this approach to record protein turnover dynamics, cells are exposed to heavy SILAC medium containing isotope-labeled lysine and arginine for several hours. At the end of the pulse-SILAC period, peroxidase labeling is conducted to biotinylate all proteins near the enzyme, including both old and newly synthesized proteins (Fig. 1*C*). The biotinylated proteins are then enriched and subjected to analysis using LC-MS/MS. Prox-SILAC expands the scope of protein turnover dynamics by capturing subcellular influences, while its operational simplicity enhances its potential applications compared to traditional fractionation methods. In a separate study, Narendra *et al.* coupled APEX2 labeling with multi-isotope imaging mass spectrometry (MIMS) to quantify the turnover of lysosomal proteins, unveiling significant heterogeneity in protein age within lysosomes (59).

### Identification of Labeled Proteins at Single-Amino-Acid Resolution

In conventional MS/MS analysis utilized in most published PL studies, the detection of “non-biotinylated peptides” from streptavidin bead-enriched proteins may generate false positives due to non-specific protein binding to the beads. To overcome this limitation, methods have been developed for directly detecting labeled peptides *via* MS/MS. For example, Spot-BioID, developed by Lee *et al.*, identifies biotin-labeled peptides through optimizing streptavidin enrichment process and detecting biotin-attached modification on lysine residue (mass shift of 226 Da) (60). However, the high affinity between streptavidin beads and biotinylated peptides has hindered the identification of labeled peptides. One solution to this obstacle is weakening the interaction. Lee *et al.* achieved this by substituting the biotin moiety in biotin-phenol substrate with the lower-affinity desthiobiotin (Spot-ID), enabling more efficient recovery and stronger MS signals of the labeled peptides (61). Similarly, replacing streptavidin with anti-biotin antibody has increased the number of identified biotinylation sites by over 30-fold (62). An alternative approach is to incorporate a bioorthogonal enrichment handle, such as an alkyne moiety, into the APEX substrate instead of biotin (63). Alkyne-labeled proteins can then be conjugated with photocleavable affinity tags, thereby enhancing the recovery and identification of labeled peptides.

In addition to reducing false positives, another advantage of detecting labeled sites lies in its ability to provide information on protein structural accessibility to PL, which facilitates annotating membrane protein topologies, mapping protein complex conformations, and identifying proximal drug-binding residue (61, 64, 65). For instance, by employing mitochondrial matrix- and intermembrane space-localized APEX2, Spot-ID generated a comprehensive map of inner mitochondrial membrane protein topology (61). Overall, although labeled site detection might have limited coverage compared to conventional PL methods owing to the low abundance of modified peptides, this strategy offers higher specificity and richer functional insights.

## Spatially Resolved Interactome Mapping

### Mapping Protein–Protein Interactions

**Fig. 2:**
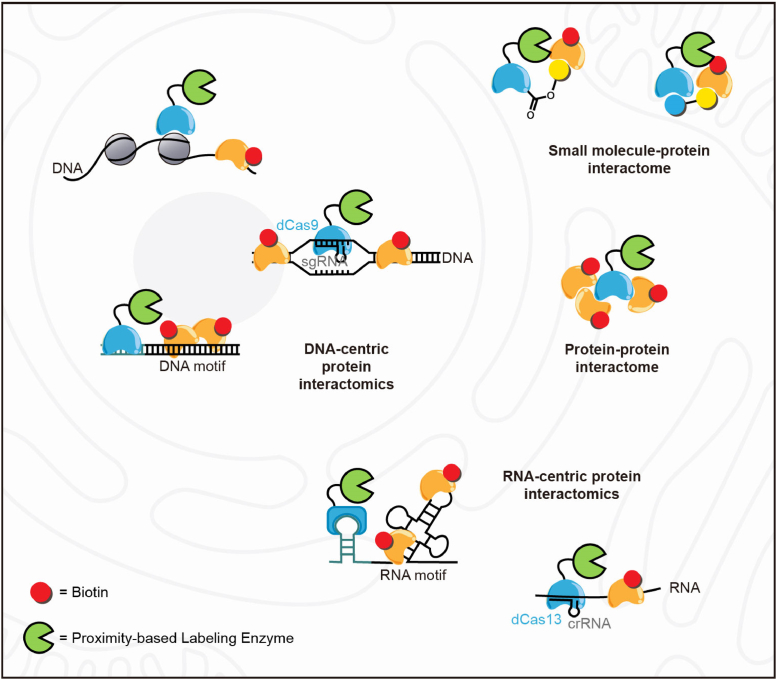
**Spatially resolved interactome mapping using PL**. The schematic illustrates representative PL-based techniques for mapping the interactomes of proteins, DNA, RNA and small molecules. In general, a PL enzyme (*green*) is genetically fused to a targeting motif that localizes the enzyme to the biomolecule of interest. Proteins surrounding the biomolecule were then biotinylated for enrichment and unbiased identification by mass spectrometry.

Investigating protein–protein interactions (PPIs) has always been a central application of PL methods and has been extensively discussed in past reviews (66, 67, 68). A notable recent advance involves the combination of APEX labeling and cross-linking mass spectrometry (CXMS) for elucidating subcellular interactomes with high spatial resolution (Fig. 2). This integrative approach, known as APEX-CXMS, leverages the rapid biotinylation by APEX and efficient protein-protein interaction profiling by crosslinking, allowing spatially-specific isolation and identification of protein interaction network in living cells by unbiased mass spectrometry analysis (69). The authors demonstrated high-throughput profiling of subcellular location-specific PPIs, identifying >600 pairs of mitochondrial PPIs and >300 pairs of nuclear PPIs, many of which were not characterized previously. Future application of this approach to various non-membrane enclosed organelles, such as stress granules or post-synaptic densities, would likely uncover novel biological insights into the molecular organization of these subcellular regions.

### RNA-Centric Protein Interactomes

The intricate interplay between proteins and RNA within cells plays a pivotal role in a wide array of cellular processes, encompassing transcription, translation, and responses to cellular stress (70, 71). Recent proteomic profiling studies across different cell lines have identified over 2000 candidate RNA-binding proteins (RBPs) in mammalian cells, representing up to 10% of the human proteome. However, to obtain a more comprehensive picture of RNA-protein interactions inside living cells, the development and application of spatiotemporally resolved mapping tools are essential. These approaches not only delineate which proteins interact with RNA molecules but also provide insights into the precise cellular locations and environmental conditions under which these interactions occur.

To resolve RBPs with subcellular precision, the Ting lab developed APEX-PS (19), a technique that combines peroxidase-catalyzed PL with organic-aqueous phase separation of crosslinked protein-RNA complexes (72, 73). APEX-PS generated a comprehensive dataset of RBPs localized in the nucleus, nucleolus, and outer mitochondrial membrane (OMM), providing a rich resource to explore the intricate pattern and physiological function of RNA-protein interactions. Notably, APEX-PS unveiled the pivotal role of SYNJ2BP, an OMM RBP, in retaining specific nuclear-encoded mitochondrial mRNAs at the OMM during translation stress. This retention mechanism facilitates localized translation and subsequent import of genomic DNA-encoded mitochondrial proteins into the mitochondrion, thus functioning as a potential mechanism during cellular stress recovery.

In addition to profiling subcellular RBPs, PL tools could also be adapted in an RNA-centric approach, for studying proteins interacting with a specific RNA molecule. The development of RaPID (RNA–protein interaction detection) facilitates the study of proteins that bind to a specific segment of RNA (74). This is achieved by appending BoxB stem-loops to both sides of the target RNA, allowing a λN-biotin ligase fusion protein to tag neighboring proteins that bind to this RNA segment (Fig. 2). Similarly, RNA-BioID employs MS2 aptamers as RNA tags, which can be specifically recognized by MS2 coat protein (MCP) (75). By inserting MS2 sequences into the 3′ UTR of the target mRNA, a fusion of MCP with BirA∗, the biotin ligase, enables tagging of proteins bound to the specific mRNA. Other strategies involve the fusion of biotin ligase with enzymes possessing programmable RNA targeting capabilities, achieving targeted tagging across native cellular RNAs without sequence modification. CARPID (CRISPR-assisted RNA–protein interaction detection) (76) and CBRPP (CRISPR-based RNA proximity proteomics) (77) takes advantage of CRISPR-Cas13 systems to precisely target an RNA of interest, using a nuclease-dead version of Cas13 (dCasRx or dCas13b) fused to biotin ligases to label and identify proteins that interact with specific RNAs within the native cellular context (Fig. 2). The successful application of these tools in identifying the interactome of long non-coding RNAs (lncRNAs) and mRNAs showcase the potential of biotin ligase-based PL in unraveling the dynamic and complex RNA-protein interactions.

Additionally, to further improve the temporal resolution of such RNA-protein interaction mapping, tools based on engineered peroxidases have also been developed to take advantage of the rapid labeling kinetics of APEX. In one interesting example, a sequence non-selective double-stranded RNA binding domain (dsRBD) was fused to dCas13 to specifically enhance its binding to the RNA duplex formed between the guide RNA and a cognate target RNA (78). The fusion of APEX2 with either MCP or dCas13d-dsRBD facilitates labeling of proteins surrounding targeted RNAs, offering a versatile platform for RNA-protein interaction mapping (Fig. 2). Benefiting from the rapid temporal response of APEX2, both MCP-APEX2 and dCas13d-dsRBD-APEX2 are well-suited for investigating dynamic processes compared to biotin ligase-dependent approaches (78, 79).

### DNA-Centric Protein Interactomes

The primary mechanism of gene expression regulation involves changes in protein partners that interact with the gene regulatory elements. Therefore, mapping the proteome associated with specific genomic regions is key to unraveling the intricate mechanisms underlying gene expression and regulation. Similar to RNA-centric protein mapping, methods for DNA-centric interaction profiling have centered on combining PL with CRISPR-based precise targeting.

The first attempt at this, termed CasID, employs the programmable DNA-binding protein dCas9 in combination with the promiscuous biotin ligase BirA∗, achieving targeted biotinylation of proteins proximal to specific DNA sequences (80). Subsequent improved versions of CasID (81, 82, 83) use dCas9 to localize peroxidase-based PL enzymes such as APEX2 to specific DNA sequences, for labeling and identification of proteins near specific DNA sequences (Fig. 2). Alternatively, the PROBER (proximal biotinylation by episomal recruitment) strategy inserts short DNA sequence of interest between tandem repeats of GAL4-binding upstream activation sequence (UAS) to construct high-copy episomes, coupled with the fusion expression of BASU and GAL4 (Fig. 2) (84). This setup enables the biotinylation of interacting proteins bound to the DNA region by BASU when such proteins engage with the DNA region of interest. In addition, to targeting selective chromatin marks, engineered chromatin readers (eCRs) were developed and characterized to assess their localization and binding preferences before generation of an eCR-BASU fusion enzyme for labeling chromatin modification-specific proximal proteomes (Fig. 2) (85). The utility of eCR-BASU fusion is elegantly demonstrated by comprehensive profiling of the proteomic landscape surrounding DNA methylation and histone tri-methylation sites (H3K4, H3K9, and H3K27) in mouse embryonic stem cells.

### Small Molecule-Protein Interactome

An emerging area where PL has just started to be applied is the identification of small molecule-protein interaction. PROCID (proximity-based compound-binding protein identification) fuses TurboID to HaloTag and employs small-molecules modified with HaloTag ligands to achieve labeling of the drug-interacting proteome (Fig. 2) (86). A similar approach, Drug-ID, utilizes SNAP-tag to generate a direct covalent linkage between drugs and the biotin ligase variant BASU for targeted biotinylation and identification of specific drug-binding proteins (Fig. 2) (87). These approaches have been extensively validated in identifying known drug-protein interactions and show great promise in discovering uncharacterized drug interactions. However, two limitations still remain: first, current methods require installation of bulky and complex reactive groups onto the small-molecule of interest, which could potentially disrupt its native interactions. Second, the labeling radii of biotin ligase and peroxidase-based proximity reactions, which are generally sufficient to explore protein interaction networks, might require further optimization for drug-protein interaction mapping. As small-molecules have finely tuned interaction networks, a smaller radius of PL is necessary. Future work leveraging novel labeling techniques, such as μMap (22) that utilizes reactive carbene intermediates, is needed to characterize drug–protein interaction at higher resolution (65, 88).

## Opto-Proximity Labeling

Although peroxidase- and biotin ligase-based PL methods are widely used for profiling subcellular proteomes and protein-protein interactions, they face challenges such as cellular toxicity from hydrogen peroxide (*e.g.*, APEX2) and limited temporal resolution (*e.g.*, TurboID), which restrict their application in complex physiological settings. To address these limitations, recent advances have introduced photoactivatable PL methods, collectively referred to as optoPL in this review. OptoPL leverages light to activate either a caged PL enzyme or photosensitive molecules, allowing for PL with greater temporal precision. OptoPL methods are categorized into three groups based on catalysts used: light-gated PL enzymes, protein photocatalysts, and synthetic small-molecule photocatalysts.

### Light-Gated PL Enzymes

The most prominent example of this category is light-gated TurboID. Since endogenous biotin is present in most organisms, TurboID may be activated before the addition of exogenous biotin, leading to background signals and reduced temporal specificity. To address this, two light-gated versions of TurboID, called photoTurbo (18) and LOV-Turbo (89), have been developed. Both variants are engineered to have minimal background activity in the dark, while biotin ligase activity is restored upon light exposure.

**Fig. 3:**
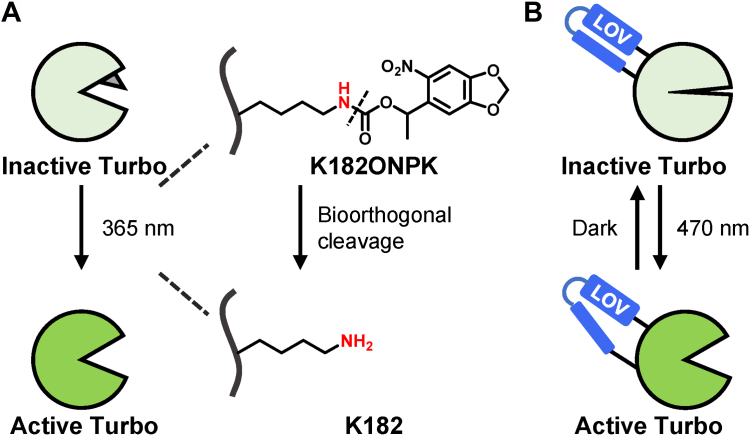
**Opto-proximity labeling with light-gated Turbo enzyme**. *A*, schematic illustration of photoTurbo activation mechanism. *B*, schematic illustration of reversible LOV-Turbo activation.

In photoTurbo, biotin ligase activity is inhibited by replacing the key catalytic lysine residue K182 with a nitrobenzyl-caged lysine *via* genetic codon expansion strategy. Upon UV-induced photolysis, the caged lysine reverts to its native form, activating biotinylation (Fig. 3*A*) (18). In LOV-Turbo, a photosensitive LOV domain is integrated into a surface-exposed loop of TurboID. The LOV domain distorts the enzyme's substrate-binding pocket through its C-terminal Jα helix. Blue light illumination releases the Jα helix, restoring the enzyme's structure to its native conformation (Fig. 3*B*) (89). Owing to its fast kinetics and reversibility, LOV-Turbo has been used for pulse-chase labeling, enabling the study of protein trafficking between organelles during cellular stress (89).

### Protein Photocatalysts

**Fig. 4:**
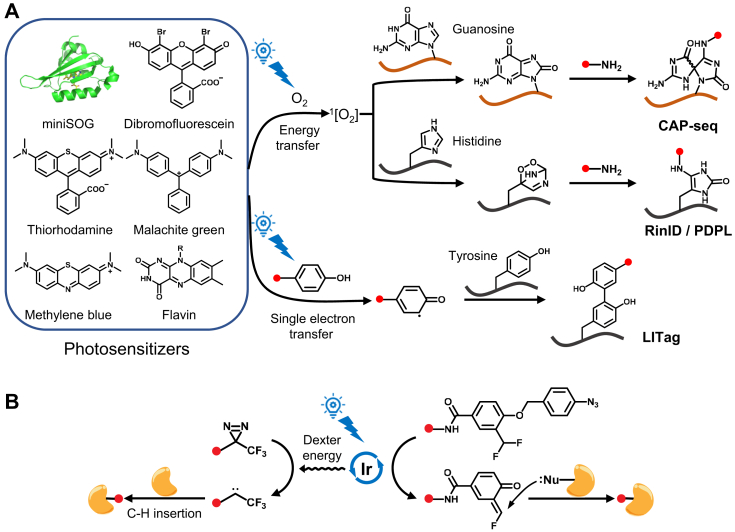
**OptoPL with genetically encoded and small-molecule photocatalysts**. *A*, mechanisms of photosensitizer-mediated optoPL towards guanosine in RNAs, and histidine and tyrosine residues in proteins. *B*, schematic illustration of the μMap (*left*) and CAT-Prox (*right*) strategy.

Protein photocatalysts are activated by visible light to generate reactive intermediates, which label nearby biomolecules. MiniSOG (mini-singlet oxygen generator), a flavin-binding protein derived from *Arabidopsis* phototropin 2, produces singlet oxygen upon blue light exposure *via* an energy transfer pathway (90). Initially designed to catalyze the polymerization of diaminobenzidine for electron microscopy, miniSOG has been repurposed as a genetically encoded tool for proximity-dependent labeling of subcellular RNAs. The singlet oxygen generated by miniSOG oxidizes guanosine nucleobases in proximal RNAs, allowing their capture by nucleophilic amine groups (91) (Fig. 4*A*). This technique, called CAP-seq (chromophore-assisted proximity labeling and sequencing), has been used to investigate the subcellular transcriptomes of compartments such as mitochondrial matrix, ER membrane, OMM, and stress granules (91, 92). MiniSOG-mediated photo-oxidation can also be applied to DNA (93). A variant with enhanced singlet oxygen quantum yield, SOPP2 (94), has been used to map lamina-associated domains when targeted to the nuclear lamina (93).

Beyond nucleic acid labeling, miniSOG-mediated PL has been extended to protein labeling (95, 96, 97). Locally generated singlet oxygen upon blue light illumination oxidizes neighboring amino acid residues, primarily histidine. The imidazole ring of histidine can be converted to 2-oxo-imidazole, which can then be labeled by nucleophilic aniline (photoactivation-dependent proximity labeling, PDPL) (95) or primary amine probes (reactive oxygen species induced protein labeling and identification, RinID) (96) (Fig. 4*A*). The labeled proteins are subsequently captured *via* affinity purification and identified by MS. With an action radius of ∼70 nm (98), miniSOG-mediated protein labeling allows for precise profiling of interactomes, such as those of the transcriptional activator BRD4 and the E3 ligase Parkin (95). Moreover, RinID’s temporal control enables pulse-chase labeling in the ER lumen, revealing different turnover rates between secreted and ER-resident proteins (96). Notably, Muir and co-workers demonstrated that SOPP (99), another miniSOG variant, could label tyrosine residues in proteins *via* a single-electron transfer mechanism using a biotin-phenol probe (Fig. 4*A*) (97).

### Synthetic Small-Molecule Photocatalysts

In addition to protein photocatalysts, various synthetic organic chromophores—such as dibromofluorescein (100, 101), thiorhodamine (102), methylene blue (102, 103), malachite green (104, 105), and flavin (106, 107)—can act as photocatalysts to label nearby proteins and nucleic acids (Fig. 4*A*). Like miniSOG, most of these small molecules are excited by visible light, generating singlet oxygen *via* energy transfer. Singlet oxygen then oxidizes adjacent biomolecules, which can be covalently tagged by nucleophilic probes for subsequent enrichment and identification. For example, thiorhodamine conjugated with antibodies or drugs, combined with a hydrazide probe (LUX-MS), has been used to map the nanoscale organization of surfaceome receptors and intercellular signaling networks within immune synapses (102). Additionally, excited chromophores can convert nearby small-molecule probes into highly reactive free radicals, which directly conjugate with biomolecules. Chen and co-workers showed that mitochondria-targeted rhodamine 123 can convert aryl azides to triplet nitrenes *via* energy transfer, allowing spatially restricted labeling of the mitochondrial proteome (108).

Transition metal complexes have recently emerged as powerful photocatalysts for PL. In 2020, MacMillan and coworkers introduced the μMap platform for mapping cell surface microenvironments. This method uses an iridium (Ir) complex excited by blue light to convert diazirines into reactive singlet carbenes through Dexter energy transfer (Fig. 4*B*) (22). Due to their very short half-life (<1 ns), these carbenes selectively crosslink with nearby biomolecules, labeling only proteins within nanometers from the Ir complexes. By using antibody-conjugated Ir catalysts, μMap has accurately identified protein compositions in the PD-L1 microenvironment and immunosynaptic junctions (22). To overcome the limited tissue penetration of short-wavelength light, red-light-excited photocatalysts such as Sn^IV^-chlorin (109) and osmium complexes (110) have been developed. These catalysts produce reactive aminyl radicals or triplet nitrenes from aryl azides, allowing for rapid tagging of nearby biomolecules. μMap and its derivative techniques have been applied to various areas, including identifying sialylated glycoproteins (23), profiling drug targets (65, 88), tracking chromatin state changes (111), and studying stress granule disassembly (112).

Another notable method is CAT-Prox (113), and its enhanced version, CAT-S (114), developed by Chen and co-workers. These methods utilize a unique photo-decaging mechanism. The mitochondria-targeting Ir complex catalyzes the conversion of aryl azide to aniline upon blue light irradiation, followed by the rapid unmasking of azidobenzyl-caged quinone methide (QM) or thioQM, which act as highly reactive Michael acceptors for protein labeling (Fig. 4*B*). CAT-S, being non-genetic, allows for quantitative analysis of mitochondrial proteome alterations in dysfunctional tissues, revealing dysregulated lipid metabolism in diabetic mouse kidneys (114).

The spatial scale of PL is determined by the diffusion radii of reactive intermediates, which depend on their lifetime and diffusion coefficient. This property can be leveraged to achieve PL across a wide range of spatial scales. Notably, the distinct diffusion radii of singlet carbene (∼54 nm), triplet nitrene (∼119 nm), and phenoxyl radical (∼269 nm) allow for multi-scale PL with adjustable labeling ranges using different probes (22, 109, 110, 115). This approach is not limited to transition-metal-catalyzed labeling but also applies to organic photosensitizers. Wells and co-workers demonstrated this by using the organic photocatalyst Eosin Y to activate distinct photoprobes, enabling the labeling of partner proteins at varying distances (116). Moreover, adjusting the structure of aryl azide probes, such as by varying PEG linker lengths, provides additional control over spatial resolution in PL (115).

## Immuno-Proximity Labeling

Conventional enzymatic PL methods typically require genetic manipulation to fuse PL enzymes with the protein of interest (POI), limiting their use in native tissues and clinical samples. Furthermore, enzyme fusion may disrupt the localization and function of the bait protein, creating additional challenges. Some targets, such as proteins with specific PTMs (*e.g.*, phosphorylated Tau), are particularly difficult to target using traditional fusion strategies. Immuno-proximity labeling (immunoPL) methods have addressed these limitations, enabling PL in primary cells and tissues without genetic manipulation.

### Protein-Centric ImmunoPL

In 2008, Honke and co-workers introduced EMARS (enzyme-mediated activation of radical sources), an antibody-directed PL method designed to study the molecular structure of the cell surface in living cells (117). EMARS used HRP-conjugated antibodies targeting cell surface receptors to catalyze the conversion of aryl azide probes into nitrene radicals, which labeled biomolecules within a limited range of 200 to 300 nm (112). Later, SPPLAT (selective proteomic proximity labeling assay using tyramide) adopted a similar approach but used cleavable biotin-tyramide probes instead of aryl azides to profile proteomes near B cell receptors (118). However, the limited membrane permeability of antibodies confines antibody-directed PL to the cell surfaces in living cells (117, 118, 119).

**Fig. 5:**
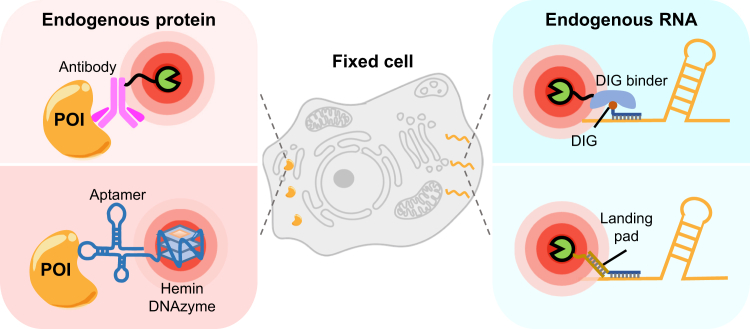
**Immuno-proximity labeling methods**. *A* and *B*, schematic illustration of antibody-targeted (*A*) or aptamer-guided (*B*) PL methods to profile biomolecules proximal to the endogenous protein baits. *C* and *D*, schematic illustration of modified oligonucleotide-directed PL techniques to probe microenvironments proximal to endogenous RNA baits.

In fixed cells and tissues, immunoPL has been successfully applied to various subcellular compartments (Fig. 5*A*). Collins and co-workers employed a primary antibody targeting Lamin A/C, followed by an HRP-conjugated secondary antibody to localize HRP to the nuclear lamina. In the presence of hydrogen peroxide and biotin phenol probes, proteins near Lamin A/C were covalently tagged and identified *via* MS. This antibody-guided PL technique, termed BAR (biotinylation by antibody recognition), was applied to primary human muscle and adipose tissues, revealing stress- and mutation-induced changes in the Lamin A/C interactome (120). Belmont and co-workers used a similar approach to map 3D genome organization relative to nuclear speckles (121) and to determine the protein composition of these speckles (122). More recently, antibody-directed PL has been used to explore the axon initial segment proteome, identifying SCRIB as a novel protein component (123). Additionally, protein A-conjugated TurboID (124) or APEX2 (125, 126) have been employed as alternatives to HRP-conjugated antibodies to map interactomes of nuclear proteins and histone modifications. A direct comparison between antibody-HRP and protein A-APEX2 conjugates targeting the H3K27me3 modification showed similar labeling efficiency, but protein A-APEX2 produced significantly lower background signals (125).

While biotin ligases have been widely used in living organisms, their application has been limited by the need to create transgenic models. Recently, Hall and co-workers introduced a GFP-directed PL method termed BLITZ (biotin labelling in tagged zebrafish) to identify interactors of cavin proteins in skeletal muscle in live zebrafish (127). Instead of directly tagging the POI, BLITZ fuses TurboID with a GFP-binding nanobody (GBP), allowing it to target GFP-fusion proteins. This approach takes advantage of existing GFP-tagged transgenic zebrafish lines, providing a less labor-intensive approach to proteomic mapping across various tissues *in vivo*. The GBP-TurboID strategy has also been successfully applied to identify tissue-specific centriolar components in *C*. *elegans* (128) and to map the interactomes of SARS-CoV-2 viral proteins (64).

Aptamers, nucleic acids that mimic antibodies, can recognize and bind specific biological targets, ranging from small molecules to cells. Compared to antibodies, aptamers offer several advantages, including ease of evolution and modification, low production costs, and reduced batch-to-batch variability (129). Recently, Yang *et al.* and Tanner *et al.* reported fully nucleic acid-based PL platforms (130, 131). In these methods, DNA aptamers targeting specific proteins are coupled with an evolved hemin-binding, peroxidase-mimicking DNAzyme (Fig. 5*B*). The aptamer-DNAzyme conjugate was used to profile cell surface proteins.

### RNA-Centric ImmunoPL

While most PL methods in fixed samples focus on proteins, advancements in RNA *in situ* hybridization (ISH) techniques have facilitated the mapping of biomolecules near specific RNAs. In 2022, Makeyev and co-workers introduced the Hypro (hybridization-proximity) technique, which systematically identifies proteomes and transcriptomes near nuclear RNA compartments (132). In HyPro, digoxigenin-modified antisense oligonucleotides hybridize to the RNA of interest, followed by incubation with a recombinant APEX2-DIG10.3 fusion protein that binds digitogenin, thereby targeting APEX2 to the RNA (Fig. 5*C*). Alternatively, Shechner and co-workers developed the O-MAP (oligonucleotide-mediated proximity-interactome mapping), a technique that employs sequential oligonucleotide hybridization to guide peroxidases to the RNA of interest (133). In O-MAP, primary oligonucleotide probes containing universal "landing pad" sequences hybridize to the target RNA, followed by attachment of HRP-conjugated secondary probes to the landing pad (Fig. 5*D*). Both HyPro and O-MAP methods have uncovered previously unknown spatial neighbors of nuclear non-coding RNAs, such as *XIST*, 45S pre-rRNA, and *NEAT1*, providing new insights into the molecular organization within the mammalian nucleus.

### Summary and Outlook

To summarize, recent advances in PL techniques have extended beyond simple protein abundance measurements to provide valuable insights into subcellular PTMs, protein trafficking, turnover, and biomolecular interactions. Additionally, optoPL and immunoPL methods complement existing enzyme-mediated PL approaches, such as APEX2 and TurboID. Given the multitude of PL tools available, one may ask: how to choose the appropriate technique? The answer depends on the sample type (*e.g.*, cell culture vs. tissue), the feasibility of genetic manipulation (*e.g.*, exogenous gene expression vs. endogenous targets), the need for high temporal resolution, sensitivity to light, and most importantly, the specific biological question being addressed.

Each PL catalyst has its strengths and limitations. Peroxidases like APEX2 (12) and HRP provide high temporal resolution for both protein and RNA labeling, with labeling time as short as 1 minute. However, their use *in vivo* is constrained by the toxicity of hydrogen peroxide, and HRP is further limited to the secretory pathway and extracellular applications. In contrast, TurboID (10) is non-toxic and has already been used to profile the cellular secretome *in vivo*, identifying tissue-specific secreted proteins in various organisms, including fruitfly and mice (39, 40, 41, 42, 43). A recent study even mapped protein secretomes across 21 cell types and 10 tissues in mice (134). However, the presence of endogenous biotin complicates temporal control of the TurboID reaction. For experiments requiring high spatiotemporal resolution, optoPL methods are ideal, provided that light irradiation is feasible. Ultimately, the choice of catalyst depends on the specific requirements of the experiment, particularly *in vivo* applicability and labeling speed. Developing more orthogonal PL enzymes remains a key goal for future research.

Notably, Dickinson and coworkers recently introduced a novel RNA PL method, BAP-seq (bioorthogonal acylating agents for proximity labelling and sequencing), which operates without generating radicals (135). This approach uses acylating agents masked by bioorthogonal 1-methylcyclopropyl (mCP) esters, which are resistant to endogenous esterases in human cells but can be rapidly hydrolyzed by an exogenously expressed *Bacillus subtilis* esterase (BS2). By controlling the subcellular expression of BS2, highly reactive acid chlorides or aryl thioesters are generated only in specific areas of interest, revealing RNA distribution across subcellular compartments, including the nucleus, cytosol, mitochondrial matrix, and nucleolus. With its bioorthogonality, rapid kinetics, and independence from light or oxidants, BAP-seq offers distinct advantages over existing PL methods. Its extension to protein PL is also a promising future direction, given the high reactivity of acid chlorides for protein acylating.

Beyond using antibodies to target PL catalysts to specific antigens, as in immunoPL, other recognition mechanisms, such as receptor-ligand interactions, can also recruit PL enzymes. For instance, fusing glucagon-like peptide 1 (GLP-1) with APEX2 allows the investigation of native membrane interaction networks for the GLP-1 receptor during agonist stimulation in live cells (136). Future developments in PL-based profiling of receptor-ligand interactions could benefit from advances in nanobody engineering and the conjugation of light-activated photocatalysts for spatiotemporal control of PL.

PL techniques have also advanced our understanding of intercellular interactions, particularly in the immune microenvironment. Techniques based on sortase (137, 138, 139), fucosyltransferase (140), and QM-decaging (141, 142), have been used to study cell-cell interactions in various immune cells. However, most of these techniques are limited to *ex vivo* studies. Developing *in vivo*-compatible PL methods would significantly enhance research in this area. For instance, recent progress in tyrosinase-based PL has enabled mapping of extracellular protein interactions in living mice (143, 144), a technique that could be adapted to study intercellular interactions *in vivo*.

Current PL methods require targeting catalytic moieties (enzymes or small molecules) to specific subcellular regions using either genetic fusion or antibodies. However, these approaches are limited when subcellular compartments lack distinct markers and can only be identified by morphological features. To overcome this challenge, Liao and co-workers developed a microscopy-guided subcellular proteomics technology, called optoproteomics. This technique uses automated microscopy to coordinate fluorescence imaging with targeted photolabeling of proteins in specific subcellular regions (145). To obtain sufficient biotinylated proteins for LC-MS/MS analysis, imaging-labeling cycles must be repeated across multiple fields of view. Optoproteomics has already identified novel proteins localized to stress granules and amyloid-β plaques with high sensitivity, specificity, and spatial resolution. Future advancements in optoPL and super-resolution imaging are expected to enhance the efficiency and precision of microscopy-guided subcellular proteomics.

## Conflict of interest

The authors declare that they do not have any conflicts of interest with the content of this article.
